# Associations between biosecurity and outbreaks of canine distemper on Danish mink farms in 2012–2013

**DOI:** 10.1186/s13028-015-0159-2

**Published:** 2015-09-30

**Authors:** Louise Gregers-Jensen, Jens Frederik Agger, Anne Sofie Vedsted Hammer, Lars Andresen, Mariann Chrièl, Emma Hagberg, Mette Kragh Jensen, Mette Sif Hansen, Charlotte Kristiane Hjulsager, Tina Struve

**Affiliations:** Department of Veterinary Disease Biology, Faculty of Health and Medical Sciences, University of Copenhagen, Ridebanevej 3, 1870 Frederiksberg C, Denmark; Department of Large Animal Sciences, Faculty of Health and Medical Sciences, University of Copenhagen, Grønnegårdsvej 8, 1870 Frederiksberg C, Denmark; National Veterinary Institute, Technical University of Denmark, Bülowsvej 27, 1870 Frederiksberg C, Denmark; Kopenhagen Diagnostics, Kopenhagen Fur, Langagervej 60, 2600 Glostrup, Denmark

**Keywords:** Canine distemper virus, Biosecurity, Mink, *Neovison vison*

## Abstract

**Background:**

During 8 months from July 2012 to February 2013, a major outbreak of canine distemper involving 64 mink farms occurred on the Danish peninsula of Jutland. The canine distemper outbreak was associated with exposure of farmed mink to infected wild carnivores and could represent a deficit in biosecurity on the mink farms. The aim of this study was to investigate the extent and association of specific biosecurity measures with the outbreak. The study was carried out in an epidemiological case–control design. The case group consisted of the 61 farms, which had a confirmed outbreak of canine distemper from July 2012 to February 2013. The control group included 54 farms without an outbreak of canine distemper in 2012 or 2013, selected as the closest geographical neighbour to a case farm.

**Results:**

The results showed that significantly more control than case farms had vaccinated their mink against canine distemper virus. Mortality was only assessed on the case farms, and there was a non-significantly lower mortality on vaccinated farms than on the non-vaccinated farms. Furthermore, the proportion of farms with observations of wild red foxes (*Vulpes vulpes*) inside the farm enclosures were larger for case farms, indicating that the control farms had a better biosecurity or were not equally exposed to canine distemper virus. Generally, all farms had very few specific precautions at the gate entrance in respect to human visitors as well as animals. The use of biosecurity measures was very variable in both case and control farms. Not using plastic boot covers, presence of dogs and cats, presence of demarcated area for changing clothes when entering and leaving the farm area and presence of hand washing facilities significantly lowered the odds of the farm having a canine distemper virus outbreak.

**Conclusions:**

The results of the study indicate that consistent use of correct vaccination strategies, implementation of biosecurity measures and limiting human and animal access to the mink farm can be important factors in reducing the risk for canine distemper outbreaks.

**Electronic supplementary material:**

The online version of this article (doi:10.1186/s13028-015-0159-2) contains supplementary material, which is available to authorized users.

## Background

Canine distemper virus (CDV) is classified in the Morbillivirus genus of the Paramyxoviridae family. The virus particle is enveloped and contains a single-stranded negative sense RNA genome of approximately 16 kb. CDV is one of the most important causes of infectious disease in both domestic and wild carnivores of the *Canidae* (e.g. dog, fox, and wolf) and *Mustelidae* (e.g. mink, ferret, badgers, and martens) orders [1]. CDV can also infect felids and cetaceans [2–6]. The virus is highly contagious and can cause high rates of mortality, especially in young animals. No efficient treatment is available, but prevention of canine distemper is possible and effective through vaccination [7]. The virus is shed in oral, respiratory and ocular fluids and exudates [1]. Transmission between animals is predominantly through aerosols. Transmission from contaminated environment is also possible although hampered by the fragility of the enveloped virus outside the host. Thus, CDV is easily inactivated by UV-light, heat and drying [7]. In addition to horizontal spread, vertical transmission may contribute to the persistence of virus in wild populations [8]. CDV has been a notifiable disease in Denmark since 1945 [9].

Outbreaks of canine distemper have occurred with variable frequency and severity on Danish mink farms (*Neovison vison*) [10] and CDV has also been diagnosed in wild carnivore species in Denmark including red fox (*Vulpes vulpes*), stone marten (*Martes foina*), wild ferret (*Mustela putorius*) and Eurasian badger (*Meles meles*) [11–13]. Between 2008 and 2010, there were no confirmed outbreaks of canine distemper on Danish mink farms. However, in 2011 three mink farms were confirmed positive for CDV and between July 2012 and February 2013 a major epidemic of canine distemper was recorded on the peninsula of Jutland with 64 farms found positive for CDV [8]. During the same period, diseased and dead wild red foxes were observed in the same areas. Molecular analysis revealed that the CDV isolates from such wild foxes and from infected farmed mink were closely related [8]. Thus, wild carnivores may have served as a reservoir for infection in commercial mink farms.

In general movement of live animals poses the greatest risk for spread of infectious diseases, however indirect contact via physical vectors such as people, vehicles and fomites are important as well [14].

In order to avoid the introduction and spread of CDV and other pathogens, the farms are dependant of efficient biosecurity procedures. Implementation of biosecurity measures is considered to have a positive effect on the health and productivity in animal production [15], and recently a direct quantitative effect has been demonstrated [16]. Consistency in the application of biosecurity measures is essential for the success of all types of animal production [17, 18]. However, several studies have shown that the level of biosecurity is often moderate to low irrespective of the type of animal production [15, 19–23]. Furthermore, compliance of the farmers to the implemented biosecurity measures is often poor [24–26].

In Denmark, it is mandatory to have a perimeter fence around every mink farm [27]. The purpose of this fence is to keep out wildlife and to prevent mink from escaping from the farm. Additionally, electrical wires on top of the fence are recommended for the same purposes. All Danish mink farms have mandatory consultations from their veterinarian four times a year, during which the biosecurity measures on the farm must be addressed [28]. Furthermore, Kopenhagen Fur visits all farmers to (among other things) discuss relevant biosecurity measures. In Denmark, it is not mandatory to vaccinate the farmed mink against CDV unless the farm is at the same location as a commercial pelting site [29].

The aim of this study was to investigate biosecurity practices and associations with outbreaks of canine distemper on Danish mink farms during the period 2012–2013.

## Methods

### Study design

The study was performed in a case–control design based on a questionnaire survey via personal telephone interviews with the owner or manager of the included mink farms. Four veterinarians from Kopenhagen Fur performed all interviews using the same questionnaire (Additional file 1). The questions focussed on biosecurity measures, CDV vaccination strategies, hygiene precautions for visitors, procedures for visiting other mink farms, access of domestic and wild life to the farm premises, purchase of breeding animals and equipment, and hygiene procedures for farm personnel. The interviews were performed in the fall of 2012 and throughout 2013.

A total of 64 case farms and 64 control farms were selected for the study. The case group consisted of mink farms diagnosed with CDV infection at the Danish National Veterinary Institute between July 2012 and February 2013. For each case farm a control farm was selected as the geographically closest mink farm with no record of canine distemper in neither 2012 nor 2013. None of the control farms experienced any clinical symptoms resembling those of canine distemper neither in the study period nor in the remaining part of 2013.

The vaccination strategy in three of the case farms were classified as ‘unknown’, because these farmers could not recall the name or type of vaccine used, which made it impossible to identify if CDV-antigens were included in the vaccine. Therefore, they were excluded from the study. Ten control farms were not included because the interviewers were either unable to contact the farms or because the farmers did not want to participate in the survey.

### Data management and statistical analyses

The distribution of each variable was evaluated and categorised. Vaccination strategies were divided into three groups based on their latest vaccination time point: no vaccination, summer vaccination (2012) and winter vaccination (2011/2012). Summer vaccinations includes those vaccinated in June–August 2012, winter vaccination includes those vaccinated in December 2011–February 2012. Summer vaccinations on the control farms also included farms, which vaccinated late in the season (September/October, 2011).

The questions about the personal hygiene measures regarded whether or not the farmers used plastic boot covers, coveralls, disinfecting footbath and/or a clear demarcation of farm area when entering the farm. Furthermore, the farmers were asked if they had access to handwashing facilities in close connection to the farm area. Access of other domestic animals to the farm premises was categorised as follows: was the farmer’s own dog allowed to enter the farm, and, if yes, whether the dog was regularly vaccinated against canine distemper or not. Observation of cats (feral or domestic) and wild red foxes on the farm premises was included as yes/no answers.

Information regarding purchase of breeding stock and mortality (only case farms) was extracted from the database at Kopenhagen Fur.

Data was analysed in SAS 9.4 (SAS Institute, Cary, NC, USA). Variables regarding biosecurity measures on the farm were investigated in the statistical analysis, although the case control design only allows for conclusions about associations. Analysis of variance for differences in mortality risk between vaccination strategies among case farms was done in proc glm. Associations between case or control farm status and explanatory variables were analysed by univariable logistic regression (proc genmod). The modeling procedure assumed the binomial distribution and used logit as the link function.

## Results

### Descriptive analysis

**Fig. 1:**
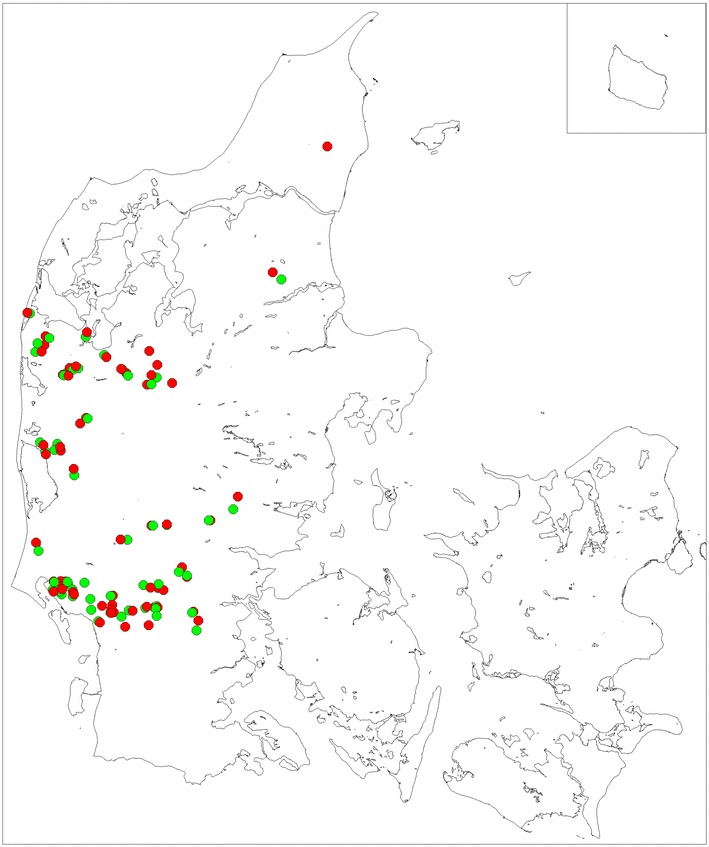
Geographical location of the 61 case and 54 control farms. Location of the farms included in the biosecurity survey. *Red circle* case farms (Farms diagnosed with CDV between July 2012 and February 2013). *Green circle* control farms (farms not diagnosed with CDV in 2012 or 2013)

A total of 61 case farms and 54 control farms were included in the analyses. Farm size varied between 240 and 21,000 breeding animals. The geographical location of the farms is illustrated in Fig. 1. The crude mortality risk on the case farms ranged between 0.004 and 29.4 %. Analysis of variance with multiple comparisons of mean mortality risk in case farms showed no statistically significant difference between the three vaccination groups (none, summer, winter). The mortality among the vaccination groups are shown in Table 1.

**Table 1:** Mortality according to vaccination strategy in 61 case farms

Vaccination (year)	Number of farms	Mean mortality	95 % confidence interval
No	14	6.1	3.0; 9.2
Summer (2012)	13	2.5	0.7; 4.2
Winter (2011–2012)	34	3.3	1.2; 5.4

Univariable analysis showed a significant effect of the following variables: Vaccination strategy, farmer visiting other farms, using plastic boot covers, hand washing facilities, use of demarcation line for changing clothes and boots when entering the mink farm area, observation of wild red foxes in farm area, access for farm dogs, cats in farm area, and purchase of breeding stock from other farms (Table 2).

**Table 2:** Results of univariable logistic regression analyses of associations between case control farm status for canine distemper virus and explanatory variables

Variable	N^a^	Cases	Controls	OR	*P* value
Vaccination^b^	115				
Yes		47	51	0.20	0.006
No		14	3	1	
Vaccination^c^	101				
No		14	3	6.46	0.017
Winter		34	19	2.48	0.08
Summer		13	18	1	
Has the farmer visited other farms?	81				
Yes		10	37	0.27	0.007
No		17	17	1	
Did visitors use plastic boot covers?	114				
Yes		39	21	2.92	0.005
No		21	33	1	
Is hand wash possible at entrences/exits?					
Yes	114	4	25	0.08	<0.0001
No		56	29	1	
Does the farm have clean and dirty zones at entrances/exits?	114				
Yes		2	9	0.17	0.020
No		58	45	1	
Were foxes observed on or around the farm?					
Yes	104	25	9	5.00	0.0003
No		25	45	1	
Did farm dogs have access to the farm area?					
Yes	91	25	32	0.13	<0.0001
No		29	5	1	
Were cats observed on or around the farm?					
Yes	114	40	47	0.30	0.012
No		20	7	1	
Has the farmer purchased or shared equipment used on other farms?					
Yes	115	4	5	0.69	0.849
No		57	49	1	
Did the farmer purchase breeding animals from other farms?	113				
No		10	18	0.41	0.043
Yes		49	36	1	

^a^Number of farmers that answered the question

^b^Vaccination categorized in yes or no regardless of season

^c^Vaccination categorized according to season of vaccination

## Discussion

Given the retrospective design of the study, time elapsed between the canine distemper outbreaks and the interviews. This could induce a recall bias, as most of the control farms were interviewed approximately 1 year after the outbreak whereas the case farms were interviewed shortly after the outbreak. However, the question causing most problems in regard to recall was whether the farmer had visited other farms prior to the outbreak. A total of 34 farmers in the case group did not answer this question. In addition, there is a potential bias in the answers to the biosecurity questions as the interviewers were employed at Kopenhagen Fur that instructs farmers in relevant biosecurity measures.

The univariable analysis showed a significant impact of the vaccination strategy implemented. Surprisingly more than 50 % of the case farms had vaccinated their animals prior to the outbreak. A possible explanation could be that most of the case farms vaccinated their mink kits earlier than the recommended 8–9 weeks of age, which could cause an interference with maternal antibodies and subsequently lead to only a partial protection against CDV infection [30]. It is also possible that some farms vaccinated to late compared to the likely time point of introduction of CDV into the farm. By vaccinating the breeding stock in winter season, mink kits born in spring would have had a period where they were susceptible to CDV; i.e. the period with insufficient protection from maternal derived antibodies until protection from vaccine-induced antibodies. The univariate analysis implies that vaccination is an important factor in protection against CDV infection, and furthermore seems to reduce the mortality on the individual mink farm when an outbreak occurs.

The use of plastic boot covers seemed to increase the odds of being a case farm. This was contrary to the expected relationship, and not in agreement with biosecurity recommendations. However, the fact that the control farms did not use plastic boot covers should not be interpreted as protective against introduction of CDV, but may instead reflect the immediate use of plastic boot covers during the outbreak in the case farms. Firestone et al. [26] recommend having a disinfecting footbath in place and changing clothes and boots when entering the farm. Racicot et al. [25] support this recommendation and state that a preferable approach is to use dedicated farm boots and having a physical barrier such as a bench, delimitating the area. Plastic boot covers are not very durable and are sometimes reused, furthermore cross contamination of personal boots or socks often occur when removing the boot covers [25]. Availability of hand washing facilities at entry and exit were also found to have a significant effect, lowering the odds of being a case farm. This measure has previously been linked with disease status on a farm [26]. However, compliance studies suggest that the presence of the facilities are not always enough to ensure that the measure is implemented [23, 25, 26].

The observation of wild foxes on the farm area was highly associated with increased odds of being a case farm. This should be regarded as a major breach of biosecurity. Additionally a recent Danish study [8] supports the assumption that wild carnivores introduced CDV to the farmed mink in 2012–2013. Therefore, the incentive to increase the general level of biosecurity to prevent disease introduction should be encouraged.

Traditionally Danish mink farms are semi-open systems with cages placed in outdoor sheds surrounded by fencing that wild carnivores can cross if no additional precautions are taken. Therefore, the farmers are encouraged to place an electrical wire on the top of the fence. The access of domestic dogs and domestic or feral cats on the farm area was significantly associated with the reduced odds of canine distemper on the farm. Data shows, that the access to the farm area of these animals was more frequent in the control farms. However, their presences are considered a breach of the general biosecurity measures.

In Denmark, it is mandatory that all animals are tested for Aleutian disease before they are moved from a farm. Therefore, Kopenhagen Fur has a database with information on movement of all live animals in Denmark. This database was used to investigate if the case and control farms had moved animals in or out of the farm prior to the outbreak. No associations were found between direct movement of animals and outbreak of canine distemper, though significantly more case farms purchased animals.

To our knowledge, this study represents the first investigation of the association between canine distemper outbreaks and biosecurity measures on Danish mink farms. A similar study has investigated biosecurity measures in association with Aleutian disease, by grouping biosecurity measures into nine variables relevant for analysis [31]. These groupings are not easily comparable since all variables regarding accessibility to the farm was grouped in one variable called “Requirements for guest’s attire” [31]. Though the farm size was found to be associated with Aleutian disease [31], the farm size was not found to be associated with outbreaks of canine distemper in this study.

## Conclusions

Vaccination against CDV is effective in protecting against disease outbreak and seems to reduce mortality if applied timely. Furthermore, clear demarcation of entry and exit area and the availability of hand washing facilities, significantly reduced the odds of a farm becoming CDV infected. The presence of foxes on/inside the farm premises significantly increased the odds of a farm becoming CDV infected.

Consistent use of biosecurity measures, limiting human and animal traffic into and out of the mink farm, and appropriate vaccination strategies are essential key factors in preventing future outbreaks of canine distemper.

## Additional file

10.1186/s13028-015-0159-2 Questionnaire (translated to English).
