# Supplementary material for: Associations between biosecurity and outbreaks of canine distemper on Danish mink farms in 2012–2013
Source: Acta Vet Scand. 2015 Sep 30;57:66. doi: 10.1186/s13028-015-0159-2 (PMC4589036; doi:10.1186/s13028-015-0159-2)
Supplement: Supplementary file 1 — 10.1186/s13028-015-0159-2 Questionnaire (translated to English). [file 13028_2015_159_MOESM1_ESM.docx]

| Farm information (fill out beforehand) | |
| --- | --- |
| Date:  (of interview) | Ph:  ( / 2014 ) Cell phone: |
| Name: |  |
| Farm no: |  |
| CHR no: |  |
| Feed supplier:  Account no: |  |
| Veterinarian:  Practice: |  |
| No of animals on farm: | Breeding:  Pups: |
| Colour type: | Scanblack, Wild type, Mahogany, White/ Pastel / Pearl |

History:

| Vaccination: | Summer: | Winter: |
| --- | --- | --- |
| Time (date):  Who vaccinated (firm?) |  |  |
| Name of vaccine: |  |  |
| Producer: |  |  |

Others animals on the farm:

| Other animals (Dog/cat):  Feral cats / other wildlife?  Fox (badger/marten/others?): |  |
| --- | --- |
| Is the dog vaccinated? (when): |  |
| Do you use the dog for hunting? Do you go hunting with other farmers? |  |

Pelting:

| Do you perform pelting yourself? If not, then whom? |  |
| --- | --- |
| Do you perform pelting for others?  If yes, for whom? (Name and address): |  |
| Will you be needing live sorting?  (KF) |  |

Visits on the farm:

| Who have you visited: (other farmers, pelting facilities) |  |
| --- | --- |
| Who has visited you in the last year (official controls/neighbours/veterinarian/others)? |  |
| When did they visit? (date) |  |
| Biosecurity in relation to visitors:  Protective suit:  Shoes:  Disinfective foot bath:  Zones: |  |

Employees on the farm:

| How many employees do you have? (Number, nationality) |  |
| --- | --- |
| Do they go home or live on the farm? |  |
| Do they have animals at home? (mink, dog, cat) |  |

Information on movements:

Purchase of animals in the last 12 months:

|  | Name and address |
| --- | --- |
| 1 |  |
| 2 |  |
| 3 |  |
| 4 |  |

Has there been other moves than the ones mentioned above?

Have you purchased any used machinery or housing?
